# Omalizumab in combination with subcutaneous immunotherapy for the treatment of multiple allergies associated with attention-deficit/hyperactivity disorder: a case report and a literature review

**DOI:** 10.3389/fphar.2024.1367551

**Published:** 2024-06-03

**Authors:** Bo Ding, Yanming Lu

**Affiliations:** Department of Pediatrics, Renji Hospital, Shanghai Jiaotong University School of Medicine, Shanghai, China

**Keywords:** omalizumab, asthma, attention-deficit/hyperactivity disorder, subcutaneous immunotherapy, case report

## Abstract

We describe the case of a 10-year-old boy with asthma (AS), accompanied by allergic rhinitis (AR), food allergy (FA), and combined attention-deficit/hyperactivity disorder (ADHD), who was treated at Shanghai Renji Hospital on 11 July 2020. The efficiency of the previous treatment with salmeterol/ticlosone was poor. Treatment with montelukast sodium resulted in development of neurological symptoms. Treatment with omalizumab in combination with subcutaneous immunotherapy (SCIT) was then initiated in our department based on anti-asthmatic therapy. Symptoms of asthma were completely controlled, and FA and AR symptoms improved. The treatment regimen led to a significant improvement in ADHD symptoms and the overall quality of life of the patient. The literature search was done in the PubMed database using “attention deficit/hyperactivity disorder/ADHD” and “asthma” as keywords, and we identified 47 relevant articles. In conclusion, our results show that treating asthma with omalizumab in combination with salmeterol/ticlosone and SCIT is efficient in controlling symptoms of multiple allergies and may lead to the improvement in ADHD symptoms and the overall quality of life of pediatric patients with ADHD. While current studies suggest that allergic diseases are closely related to ADHD, there is still a lack of studies or case reports of complete treatment protocols to provide clinical clues for management of the disease.

## Introduction

Half of the global population is expected to develop allergic diseases in 2050 (Cecchi et al., 2018; Ren et al., 2022). Asthma (AS) is one of the most prevalent childhood-onset allergic diseases that arises due to a combination of environmental and genetic factors and is manifested as a chronic inflammation state with reversible constriction of the airways (Leffa et al., 2021). Asthma is characterized by symptoms such as recurrent cough, wheezing, and shortness of breath (Yuksel et al., 2008; Sun et al., 2021) that profoundly impact patients’ quality of life.

Attention-deficit/hyperactivity disorder (ADHD) is considered the most common neurodevelopmental and behavioral pediatric disease (Zhou et al., 2019) and manifests as a high level of inattention, hyperactivity, impulsive behavior, learning disabilities, sleep disturbances, and, often, social isolation (Bonvicini et al., 2016; Chai et al., 2021). The prevalence of ADHD in children is approximately 7.2%, with 50% of cases persisting into adulthood (Developmental Behavioural Group of the, 2020).

The correlation between ADHD and inflammatory and autoimmune diseases, including asthma, has long become a focus of attention. Recent studies suggested that ADHD patients are more likely to present with asthma, allergic rhinitis (AR), allergic conjunctivitis, atopic dermatitis, and psoriasis (Wang et al., 2018a; Chang et al., 2021). Studies also show that various inflammation-associated genes and inflammatory mechanisms play a role in the occurrence of ADHD (Leffa et al., 2018; Wong et al., 2022). For example, increased interleukin-6 (IL-6) levels may impact neural pathways, neurogenesis, and synaptic plasticity in the prefrontal cortex and hippocampus of pediatric patients (Hunter and Jones, 2015). Current evidence suggests that respiratory allergic diseases have a high probability of comorbidity with eczema and food allergies (Pols et al., 2015; Esteban-Gorgojo et al., 2021; Laidlaw et al., 2021), which further increases the risk of ADHD (Shyu et al., 2012). Research also demonstrated the effectiveness of subcutaneous immunotherapy (SCIT) therapy in alleviating ADHD symptoms in children with difficult-to-treat ADHD (Yu et al., 2023).

ADHD patients with allergic diseases often have difficulties in effective self-management, which increases the complexity of allergic disease treatment, prolongs the course of the disease, reduces the quality of life, and seriously affects children’s growth and development as well as their physical and mental health (Jin et al., 2021; Ren et al., 2022). In this study, we report the case of a pediatric patient with multiple allergic diseases and ADHD, treated with omalizumab and SCIT. Additionally, we performed a literature search to identify studies that describe the connection between ADHD and allergic diseases in the pediatric population. Together with the existing literature, our observations may contribute to deepening the understanding of the clinical characteristics of allergic diseases and provide potential clinical ideas for the diagnosis and treatment of such patients.

### Case presentation

#### Patient information and medical history

We present the case of a male child, 10 years old, admitted to Shanghai Renji Hospital on 11 July 2020 because of “recurrent wheezing for 8 years,” with the diagnosis of allergic asthma, allergic rhinitis, food allergy, ADHD (combined type), and intellectual disability. Written informed consent was obtained from the patient.

The child was gravida 2 para 1, full term, delivered by Cesarean section, with no history of choking resuscitation, mixed feeding, and immunizations on time. The child had a history of recurrent eczema (disappeared at 1.5 years), was talking at 2 years, and had rhinitis at 3 years.

#### History of neurodevelopmental disorder

At 8 years, the child was diagnosed with intellectual disability and combined ADHD and was treated with methylphenidate hydrochloride, but the symptoms of ADHD were not effectively controlled. The patient did not undergo cognitive behavioral therapy (CBT). Eventually, the child discontinued the drug on his own and was not taking any ADHD medications upon admission.

#### History and symptoms of allergic disease

The patient had a family history of allergic diseases (both parents and sister are allergic). Wheezing first manifested at the age of 1.5 years. The child suffered from recurrent respiratory infections from birth to 2 years of age. Wheezing occurred over six times/year after the age of 3 and was induced by cold air, exercise, rainy weather, and emotional changes. Cough was dry, occurred in the absence of respiratory infection, was induced by cold air, and predominantly occurred at night. There was no evidence of fever, foreign body aspiration, hoarseness, chest tightness, sighing, acid reflux, belching, early satiety, night sweats, lethargy, hemoptysis, and nocturnal sleep snoring during the entire course of the illness. Asthma was accompanied by food allergies to eggs and fish (rash, itching, and cough after eating) and to azithromycin.

#### Initial presentation, diagnostic tests, and treatment

A physical examination revealed the following characteristics of the patient: height, 130 cm; weight, 30 kg; hyperactive; the nasal mucosa was slightly pale with edema; and a small amount of clear mucus; double-lung auscultation respiratory sounds were thick with mild wheezing and no wet rales. Heart, abdomen, and nervous systems were normal.

The blood routine detected the following: eosinophils (EOSs), 5.6%; total immunoglobulin E (IgE), 3,380 (IU/mL); household dust mite immunoglobulin E (IgE), 19.3 IU/mL; and mixed mold IgE, 24.3 IU/mL.

Pulmonary function tests and chest computed tomography (CT) detected no abnormalities.

#### History of treatment

At 8 years of age, the patient underwent treatment with a nebulizer containing budesonide suspension for inhalation, two times a day for 2 months, but discontinued the medication on his own after improvement. At 9.5 years of age, the patient had a severe asthmatic attack with progressive restriction of movement and recurrent episodes of wheezing. At the age of 10, treatment with inhaled corticosteroid (ICS)/long-acting beta agonist (LABA) was initiated: ICS (ticlosone; 100 mg) and LABA (salmeterol; 50 mg) inhalations. However, due to the long duration of asthma and poor ICS–LABA administration control, the treatment was not effective in controlling the symptoms. Oral leukotriene receptor antagonist (LTRA) montelukast sodium (one sachet, once a day) was added, but the patient reported a state of euphoria throughout the day, which resolved after ICS–LABA–LTRA treatment was discontinued.

**FIGURE 1:**
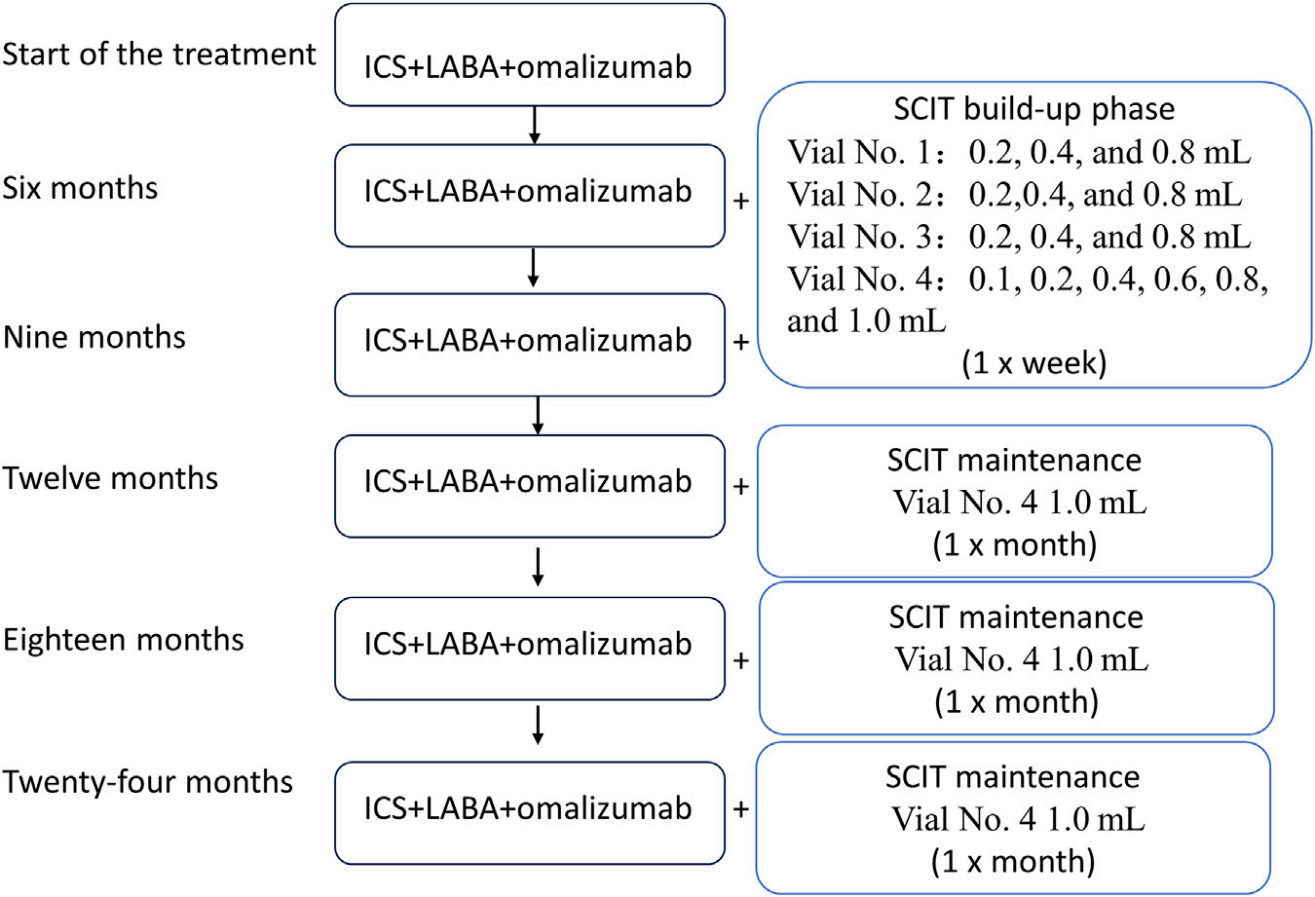
Flowchart of the treatment regimen. The patient was started on the ICS, LABA, and omalizumab treatment, and SCIT therapy was initiated 6 months after the beginning of the treatment. SCIT: subcutaneous immunotherapy; ICS: inhaled corticosteroid; LABA: long-acting beta-agonist.

After the diagnosis upon admission, the following treatment was initiated, as shown in Figure 1: a child received initial treatment with ICS (ticlosone; 100 mg) + LABA (salmeterol; 50 mg) + omalizumab (150 mg). ICS + LABA was initially used in the morning and evening, one inhalation per time; after 9 months of treatment, the ICS + LABA dosage was reduced to once a day, one inhalation per time; after 18 months of treatment, inhalations were performed every 2 days, one inhalation per time. The initial omalizumab dosage was 150 mg/month. Starting from 18 months, the dosage was changed to 150 mg/2 months up to 24 months of follow-up. The patient reported no side effects.

SCIT was initiated 6 months after the beginning of ICS/LABA/omalizumab treatment. Mite allergen-specific immunotherapy was carried out by subcutaneous injections of the high-dose house dust mite allergoid (Alutard SQ, Denmark), which is provided in the form of four injectable vials with the following concentrations: 100 SQ-U/mL for vial 1; 1,000 SQ-U/mL for vial 2; 10,000 SQ-U/mL for vial 3; and 100,000 SQ-U/mL for vial 4. The dose escalation rules were as follows:

Vial 1: 0.2 mL, 0.4 mL, and 0.8 mL (1–3 weeks).

Vial 2: 0.2 mL, 0.4 mL, and 0.8 mL (4–6 weeks).

Vial 3: 0.2 mL, 0.4 mL, and 0.8 mL (7–9 weeks).

Vial 4: 0.1 mL, 0.2 mL, 0.4 mL, 0.6 mL, 0.8 mL, and 1.0 mL (10–15 weeks).

In the first 15 weeks, the injections were administered once a week. After 15 weeks, injections were administered once a month. Each injection had a dose of 1.0 mL (Figure 1). On the 12-month visit, the patient reported wheezing after the desensitization treatment. The follow-up history revealed that ICS had been discontinued on its own. Self-discontinuation or self-reduction of medication can lead to a state of uncontrolled asthma (Rogers and Reibman, 2012). Therefore, it is plausible that due to the predominantly allergic nature of asthma, a patient who discontinues the medication on their own while being exposed to high concentrations of mite allergens during SCIT can eventually develop an acute asthma attack. The child was instructed to resume regular medication, and the symptoms resolved.

The recorded patient outcomes included subjective improvement, rhinitis symptoms, asthma symptoms, quality of life, and food allergies.

Asthma severity was assessed using the Childhood Asthma Control Test (CACT), a childhood asthma control tool with seven questions. The first four questions have a score range of 0–3, and the last three questions have a score range of 0–5, for a total score of 27. The scores were as follows: score ≤19, uncontrolled asthma; 20–22, partially controlled; and ≥23, well-controlled (Liu et al., 2007).

Additionally, asthma symptoms and allergic rhinitis symptoms were assessed using the visual analog scale (VAS) score, a subjective visual analog scale that rates rhinitis or asthma symptoms, with a score of 0 representing no symptoms and 10 representing the most severe symptoms (Bousquet et al., 2007). Symptom scores were assessed based on the severity of the main symptoms of asthma/rhinitis (asthma assesses cough, wheezing, shortness of breath, and chest tightness, and rhinitis assesses nasal congestion, itchy nose, runny nose, and sneezing). The classifications were as follows: none, no symptoms; mild, symptoms are mild and easily tolerated; moderate, symptoms are noticeable and annoying but tolerable; and severe, symptoms are intolerable and interfere with daily life or sleep (Zhonghua, 2022).

The quality of life was assessed using the Pediatric Asthma Quality of Life Questionnaire (PAQLQ), which consists of 23 questions, each rated on a scale of 1–7 (Sztafińska et al., 2017), with higher scores indicative of a better quality of life.

ADHD symptoms were assessed according to the Diagnostic and Statistical Manual of Mental Disorders—Fourth Edition (DSM-IV) (Pappas, 2006) and Swanson, Nolan, and Pelham Rating Scale (SNAP-IV-18), an 18-question reporting inventory designed to measure attention deficit/hyperactivity disorder (ADHD) (Swanson et al., 2001).

The ambient Air Quality Composite Index (AQCI) was used to assess the comprehensive condition of urban ambient air quality. The AQCI considers the pollution degree of six pollutants, namely, SO_2_, NO_2_, PM10, PM2.5, CO, and O_3_. A higher value of the AQCI indicates a heavier pollution degree.

As shown in Table 1, after 6–9 months of omalizumab treatment, AR, asthma symptoms, and food allergy were basically controlled. A 12-month follow-up showed that the child developed wheezing after SCIT treatment. Blood work (Table 2) showed a continuous marked improvement with progression of the treatment, with an almost twofold decrease in the white blood count (WBC) levels, and an eightfold decrease in the EOS levels (%) 24 months after the beginning of the ICS/LABA treatment, which corresponds to the 18 months after the beginning of the SCIT treatment. Similarly, pulmonary function tests (Table 3) demonstrated a marked improvement in the lung function of the patient.

**TABLE 1:** Assessment of symptoms.

Time	Assessment of allergy symptoms							
	SI (%)	AR (Y/N)	AR VAS	AS (Y/N)	AS VAS	AS CACT	PAQLQ	FA (Y/N)
Start of the treatment		Y	2	Y	7	15	125	Y
3 months	50	Y	1	N	0	23		Y
6 months	60	N	0	N	0	25	150	Y
9 months	>60	Y	0	N	0	23		N
12 months	>60	N	0	Y	1	23	130	N
18 months	>60	N	0	N	0	24		N
24 months	>60	N	0	N	0	23	130	N

SI: subjective improvement; AR: allergic rhinitis; AS: asthma; FA: food allergy; VAS: visual analog scale; C-ACT: childhood asthma control test; PAQLQ: Pediatric Asthma Quality of Life Questionnaire.

**TABLE 2:** Blood and pulmonary function indexes.

	WBC 10^9/L	Hgb g/L	PLT 10^9/L	EOS %	EOS 10^9/L
Start of the treatment	10.17	135	297	5.6	0.57
6 months	5.30	114	303	3.3	0.17
12 months	7.18	129	202	1.8	0.13
24 months	5.78	138	220	0.7	0.04

WBC: white blood count; Hgb: hemoglobin; PLT: platelet; EOS: eosinophil.

**TABLE 3:** Pulmonary function tests.

PFT	Time	
	Start of the treatment	24 months
FEV1(L)	1.58	2.71
VC(L)	1.88	2.97
FEV1/VC max (%)	98.6	108.2
FEV 75 (%)	77.7	106.6

FEV1: forced expiratory volume in 1 second; VC: vital capacity.

As shown in Table 4, after the treatment, ADHD symptoms and the quality of life of the patient markedly improved.

**TABLE 4:** Progression of ADHD symptoms.

Time	DSM-Ⅳ	SNAP-Ⅳ-18	Conners’ questionnaire for parents				
			Conduct issues	Learning issues	Psychosomatic problems	Impulsiveness and hyperactivity	Apprehensiveness
Start of the treatment	A: 1) 8/9; 2) 7/9; B, C	5.4	1.58	2.5	0.8	2.5	1.75
3 months	A: 1) 6/9; 2) 5/9; B, C	4	1.5	2.25	0.6	2	1.75
6 months	A: 1) 6/9; 2) 4/9; B, C	3.2	1.3	2	0	1.75	1.25
12 months	A: 1) 5/9; 2) 4/9; B, C	3	1.25	2	0	1.5	1.25
24 months	A: 1) 4/9; 2) 4/9; B, C	2.7	1.08	1.75	0	1.25	1

DSM-IV: Diagnostic and Statistical Manual of Mental Disorders–Fourth Edition; SNAP-IV: the Swanson, Nolan, and Pelham Rating Scale.

Environmental air quality was continuously monitored throughout the entire course of the treatment (Table 5), and no marked fluctuations in the levels of pollutants were detected.

**TABLE 5:** Local environmental situation during the course of the treatment

	Shanghai’s overall ranking in China	AQCI	PM2.5 (μg/m^3^)
Start of the treatment	112	3.37	27
3 months	27	3.01	19
6 months	17	4.19	39
9 months	80	3.64	31
12 months	84	2.59	17
18 months	24	3.82	42
24 months	137	3.11	22

AQCI: Ambient Air Quality Composite Index. *Data from the China Environmental Monitoring Center.

## Literature review

We searched PubMed, Embase, and Chinese Medical Association Journal full-text databases using the keywords “asthma” and “ADHD/Attention-Deficit Hyperactivity Disorder.” We also searched the Chinese Medical Association Journal Network using the keywords “asthma” and “ADHD.” The initial search identified 47 articles related to asthma and ADHD in children.

## Discussion

This study reported a case of asthma with ADHD in a pediatric patient with a poorly controlled condition who was treated with the basic ICS + LABA regimen. Our study showed that omalizumab/SCIT treatment led to significant alleviation of asthma symptoms and related allergic comorbidities and was associated with a marked improvement of ADHD in the patient. The effect of the treatment in our study was not affected by the changes in the levels of environmental pollutants.

While ICS therapy is considered a cornerstone of the pharmacological treatment of asthma (The Subspecialty Group of Respiratory DiseasesEditorial Board and Chinese Journal of Pediatrics, 2016), numerous studies emphasize the importance of the appropriate control of inhaled hormones and assessment of comorbidities in asthma patients (Tajdini et al., 2019; Eyerich et al., 2020). However, ADHD patients with allergic diseases often have difficulties in effective self-management, which increases the complexity of allergic disease treatment, prolongs the course of the disease, reduces the quality of life, and seriously affects growth and development, as well as the physical and mental health of the affected pediatric patients (Jin et al., 2021; Ren et al., 2022).

In the case described in our study, the child was initially treated with ICS + LABA + LTRA. However, due to neuropsychiatric symptoms, LTRA treatment was discontinued, and the overall achieved control of AS was poor. Omalizumab, a monoclonal antibody against human IgE, can be used for moderate-to-severe persistent allergic asthma in children 6 years and older with asthma uncontrolled by ICS + LABA therapy alone (The Respiratory AllergyAsthma Group of Chinese Thoracic Society and Chinese Medical Association, 2019). The treatment regimen of the patient in the current study was, therefore, adjusted to ICS + LABA + omalizumab, which resulted in good asthma control and significantly improved quality of life. Omalizumab can be considered a first-line treatment in patients with early-onset allergic asthma with a history of allergies and/or clinically significant IgE >100 IU/mL with allergic rhinitis and moderately elevated fractional exhaled nitric oxide (FeNO) (50 ppd) (Eyerich et al., 2020). Currently, the baseline IgE range for omalizumab treatment is 30–1,500 IU/mL. Although the guidelines do not recommend omalizumab for patients with a total IgE >1,500 IU/mL, some studies have shown that the use of omalizumab in pediatric asthma patients with high levels of IgE can help children achieve asthma control and reduce acute attacks with some degree of clinical benefit (Maselli et al., 2013; Wang et al., 2018b; Hutyrová et al., 2018; National Clinical Medical, 2021). In our study, using omalizumab resulted in rapid improvement of asthma and other IgE-mediated concomitant allergic symptoms (allergic rhinitis and food allergy) and reduced the use of hormones and associated medications (Hutyrová et al., 2018; Liu and Yin, 2019). However, it is important to note that while we reported poor AS control using the ICS + LABA regimen, this outcome may be directly related to ADHD diagnosis of the child and poor compliance with the treatment. The effect of ICS + LABA is mainly affected by two things: frequency of dosing and ability to administer the medication correctly. In this study, the child’s compliance with the doctor’s instructions for medication dosing was confirmed using a hospital case system during a follow-up consultation and by checking with the parents before administering omalizumab injection treatment. However, because of ADHD, the patient was unable to cooperate with deep inhalation of the drug and sufficient breath-holding after the inhalation. This is consistent with previous studies that showed that poor compliance with ICS + LABA (such as insufficient frequency of the treatment and poor quality of administration) may directly affect the efficiency of the treatment (Makhinova et al., 2015; Averell et al., 2022).

### Future studies are needed to validate our observations

In addition to recurrent uncontrolled asthma attacks, the patient in our study presented with allergic symptoms in infancy, multiple allergic and non-allergic comorbidities, and significantly elevated total IgE levels, and mite and mold sIgE. Studies show that the onset of allergies is closely related to exposure to allergens or environmental factors (Holgate, 1999; Kay, 2001; Galli et al., 2008). In agreement with these observations, in our study, the allergic symptoms of the patient were triggered by mites and molds, which are prevalent in the warm and humid climate of Shanghai and, therefore, make it difficult to control completely, ultimately leading to recurrent asthma attacks based on irregular asthma treatment.

Research shows that allergic asthma with allergen sensitization (mite allergen sensitization) is associated with an increased risk of ADHD (Yang et al., 2018). This association of allergic diseases with ADHD was further confirmed by the results of our literature search. However, only few studies have reported on synergistic treatments and interventions for ADHD in children with asthma. Most studies, identified by our literature search, reported on the association of ADHD with allergic diseases. Tsai et al. (2014) showed that the 1-year prevalence of ADHD peaked at the ages of 6–11 years. In addition, the prevalence of asthma was significantly higher in the ADHD group. Jiang et al. found that airway allergic disease increases the risk of ADHD in children and is an independent risk factor of pediatric ADHD (Jiang et al., 2017). Holmberg et al. demonstrated a relationship between the frequency of parent-reported asthma attacks and the probability of developing ADHD over 1 year (Holmberg et al., 2015). It was reported that AS can increase core ADHD symptoms (Lin et al., 2016), and that ADHD patients who were born premature and whose mothers experienced moderate-to-extreme stress during pregnancy had an increased risk of AS (Grizenko et al., 2015). Genetic and environmental risk factors may collectively contribute to the onset and development of ADHD (Cowell et al., 2019; Liu et al., 2019). Therefore, identifying modifiable neuropsychological risk factors for ADHD in early childhood can inform prevention strategies. Cowell et al. (2019) suggested that mother-specific responses are significantly associated with a greater risk of ADHD. Birth cohort studies have shown (Liu et al., 2019) that 2.9% of children have ADHD and that the offspring of asthmatic parents are at an increased risk of ADHD. More specifically, the offspring of mothers with AS episodes during labor and prenatal and postnatal periods are at an increased risk of ADHD (Pols et al., 2015).

Allergen sensitization may play a role in asthma and ADHD co-morbidity through neuroimmune pathways. While the exact mechanisms by which asthma influences the central nervous system (CNS) and *vice versa* are not known, there is evidence that chronic inflammatory conditions can lead to a disruption in the homeostasis of the neuroimmune environment (Konstantinou et al., 2022). Potential mechanisms associated with asthma and ADHD (Tsai et al., 2014; van der Schans et al., 2016; Chang et al., 2022; Gholami-Mahtaj et al., 2022; Jin et al., 2022) are possibly related to the effects of chronic allergic inflammation.

Numerous studies show that AS is a disease that is associated with type 2 cytokines, such as interleukin-4 (IL-4), IL-5, and IL-13, which promote AS symptoms such as airway eosinophilia, overproduction of mucus, bronchial hyperresponsiveness, and high levels of IgE (Steinke and Borish, 2001; Kuruvilla et al., 2019; Wang et al., 2023). Similarly, symptoms of AR and food and skin allergies are induced by the acute, intermittent, or chronic type 2 (T2) inflammation and secretion of pro-inflammatory cytokines and eotaxins that can cross the blood–brain barrier, resulting in neuroinflammation (Tamayo et al., 2024). Importantly, Guedes et al. (2023) demonstrated the impact of elevated IL-4 levels during cerebellar maturation and provided the first line of evidence for a mechanistical link between allergic disease and ADHD in humans. Additionally, mast cells express receptors for neuropeptides and neurotransmitters, thus playing a key role in the interplay between allergic diseases and neurological comorbidities (Greene et al., 1985; Bienenstock et al., 1988; Stead et al., 1989; Kiernan, 1990; Williams et al., 1997), while IgE high-affinity receptors and other T2 receptors are expressed on sensory (Kiernan, 1990) or enteric neurons and are functional and able to transmit signals to the CNS (van der Kleij et al., 2010; Crosson et al., 2021). Taken together, these studies suggest that controlling environmental allergen exposure may help reduce the severity of ADHD and delay the disease progression and allergen immunotherapy (Pelsser et al., 2009; Mi et al., 2020). Indeed, Yu et al. showed that SCIT therapy for AR improved ADHD outcomes in children with difficult-to-treat ADHD (Yu et al., 2023). Previous studies show that SCIT in combination with omalizumab effectively increases patient tolerance during the initial dose-escalation phase, with 90% of patients achieving a maintenance dose. Addition of omalizumab reduced the risk of local and systemic reactions associated with SCIT treatment and improved patient adherence to the treatment (Kuehr et al., 2002; Pfützner and Schuppe, 2021). Our results are consistent with these reports and demonstrate that the combination of ICS + LABA, omalizumab, and SCIT was effective in generating mite allergen immune tolerance and improving multiple allergies and ADHD-related symptoms.

In conclusion, we report a case of a pediatric patient with asthma and ADHD, who showed significant improvement in outcomes such as asthma control, associated comorbidities, quality of life, and ADHD symptoms after undergoing a treatment with omalizumab + SCIT on the basis of the ICS + LABA regimen. Taken together with the results of the literature search, our study provides some clues and a clinical basis for developing tailored treatment plans for children with allergic asthma in combination with ADHD.

## Data Availability

The original contributions presented in the study are included in the article/Supplementary Materials, further inquiries can be directed to the corresponding author.
